# Immunoglobulin VDJ repertoires reveal hallmarks of germinal centers in unique cell clusters isolated from zebrafish (*Danio rerio*) lymphoid tissues

**DOI:** 10.3389/fimmu.2022.1058877

**Published:** 2022-12-08

**Authors:** Doaa Waly, Aradana Muthupandian, Chia-Wei Fan, Harrison Anzinger, Brad G. Magor

**Affiliations:** Department of Biological Sciences, University of Alberta, BioSci Bldg, Edmonton, AB, Canada

**Keywords:** antibody affinity maturation, VDJ repertoires, germinal center, evolution, somatic hypermutation, fish

## Abstract

DNA mutagenesis during antibody affinity maturation has potentially oncogenic or autoimmune outcomes if not tightly controlled as it is in mammalian germinal centers. Cold blooded vertebrates lack germinal centers, yet have a functional Ig gene mutator enzyme, Aicda. In fish there are clusters of Aicda^+^ cells encircled by pigmented ‘melano-macrophages’ and we test the hypothesis that these clusters are functionally analogous to germinal centers. Sequenced IgH VDJ repertoire libraries from individual isolated clusters showed evidence of B-cell clonal expansion and VDJ somatic hypermutation. Construction of Ig clonal lineage trees revealed that unlike surrounding lymphoid tissue, each cluster is dominated by a few B-cell VDJ clonotypes having hundreds of mutated variants. Recruitment of B-cells to the clusters appears to be ongoing, as there are additional Ig clones having smaller lineages. Finally, we show evidence for positive selection for replacement mutations in regions encoding the antigen contact loops, but not in the framework regions, consistent with functional antibody modification. Melano-macrophages appear to trap the Ag used for post-mutation B-cell selection, performing a role analogous to the follicular dendritic cells of mammalian germinal centers. These findings provide insights into the evolution of the affinity maturation process, the improvement of fish vaccines and possibly also the workings of atypical ectopic germinal centers generated in several human diseases.

## Introduction

The germinal center (GC) process in mammals, which leads to antibody affinity maturation, has several hallmarks: 1) activated B-cells (plasmablasts) and T_H_-cells are recruited to the center by chemokines secreted by follicular dendritic cells (FDCs) and once there the B-cells (now centroblasts) clonally expand. 2) During clonal expansion activation-induced cytidine deaminase (Aicda) generates somatic hypermutations in the immunoglobulin (Ig) VDJ exon of the B-cells. 3) post-mutation B-cells (now centrocytes) undergo a selection process that favors B-cells with improved antigen binding affinities. 4) Selected centrocytes receive survival signals from FDCs and follicular T_H_-cells that rescue them from a pro-apoptotic state.

VDJ recombination, which generates part of the unique antigen (Ag) binding site for the antibodies (Abs) produced by each B-cell, also provides a unique genetic ‘fingerprint’ for each B-cell. This allows precise lineage tracking among activated B-cells (centroblasts) that are clonally expanding, and acquiring Aicda-mediated gene mutations, within the follicle/germinal center (1–3). The centroblasts, in addition to upregulating Aicda expression, also upregulate genes associated with a pro-apoptosis pathway (4). By an unknown trigger, centroblasts differentiate into centrocytes, which downregulate Aicda expression, and upregulate membrane antibody (Ab) expression. Centrocytes, which may have Ab with altered Ag binding affinities, compete for a limited number of native antigen complexes retained on the surface of follicular dendritic cells (FDCs) *via* complement receptors 1 and 2, or FcRγII. Upon binding Ag complexes, the centrocytes may also receive further co-stimulatory signals from cell adhesion molecules on the FDCs, or secreted IL-6 and BAFF. Centrocytes with high affinity for the antigen endocytose it, and present Ag peptides *via* MHC class II to follicular T_H_-cells, from which they receive further survival signals. Selected cells can revert back to the centroblast stage for more mutation and proliferation, or undergo a phase of proliferation, with daughter cells differentiating into either long-lived memory cells or antibody-secreting plasma cells. The net effect of antibody affinity maturation is a 100 to 1000-fold increase in Ab binding affinities, both of secreted Ab, and of those populating the membrane of memory B-cells. As centrocytes have a pro-apoptosis gene expression profile (4, 5) cells that fail in getting rescue signals (selection) will die off (6).

Cold-blooded vertebrates have a humoral immune response similar to that of mammals, but with fewer antibody isotypes. All studied teleost fish have IgM and IgD, which are generated by alternative pre-mRNA splicing of a common transcript, as in mammals. Additionally, several teleosts, including the Cyprinidae, have an IgT isotype (for ‘teleost’) (7, 8), which is referred to as IgZ in zebrafish. IgZ/IgT is generated during a variant form of VDJ recombination during B-cell development. In zebrafish, the IgH locus consists of several V_H_-elements, common to all isotypes, followed by Dζ, Jζ and, Cζ regions of IgZ and D, J, Cμ and Cδ regions downstream of the IgZ C region (7).

Though fish have humoral immunity, they lack both histologically distinct GCs and cells resembling FDCs (9, 10). It has been argued that these ‘lower’ vertebrates also lack Ab affinity maturation, though Ab affinities do increase 10 to 100-fold following immunization (11–13). We, and others, have identified fish homologues of Aicda, which can drive mutagenesis in murine Aicda^-/-^ B-cells, as well as in a fish B-cell line stimulated to induce Aicda expression (14–17). Using *in situ* hybridization, Aicda expressing cells were localized to clusters of auto-fluorescing melano-macrophages (MMΦs) in the spleen and kidney of channel catfish, along with cells expressing IgM heavy chain, or CD4 transcripts (16). Subsequent studies in goldfish and zebrafish (both Cyprinidae) determined that MMΦs were surrounded by interdigitating reticular cells, which are required for long-term survival (>200 days) of MMΦs in co-culture (18). Earlier studies had established that the clusters were also sites of long-term (months) retention of various Ag types, which has been viewed as analogous to FDC Ag trapping in GCs (19–21).

Here, we report the isolation of melano-macrophage clusters (MMΦCs) from zebrafish lymphoid tissues and generation of VDJ repertoire libraries. Clusters show evidence of expansion of B cell clonotypes and hallmarks of positive selection in the variable regions. We show evidence of capture of intact antigen on melano-macrophage cells. These hallmarks of germinal center responses restricted within fish melano-macrophage clusters provide important insights into the evolution and regulation of antibody diversification by Aicda.

## Materials and methods

### Melano-macrophage autofluorescence and choice of antigen fluorescent tag

We previously used laser scanning confocal microscopy emission or λ profiles to establish that channel catfish melano-macrophage pigments excited with a 488 nm laser (FITC) emit at peak of 545 to 555 nm and tail off at approximately 580 nm (16). Similar FITC λ profiles were observed for goldfish and zebrafish melano-macrophages (not shown). To determine if cyprinid melano-macrophage autofluorescence extended beyond 600 nm we excited cells with a Cy5 laser (633 nm) and observed minimal or no emission beyond 640 nm (**Figure S1A**), As such we chose Alexa-647 as a distinguishable antigen label for vaccination (**Figure S1B**). To verify that Cy5 autofluoresence did not occur in most melano-macrophages, we did FACS analysis of unvaccinated goldfish melano-macrophages from spleen and kidney. Less than 0.1% of cell events were seen in the Cy5 channel, similar to what was observed for goldfish PBLs, which lack melano-macrophages (not shown).

### Vaccination and sample preparation for sequencing

All fish maintenance conditions and procedures were approved by the University of Alberta Animal Care and Use Committee of the Research Ethics Office, according to guidelines set by the Canadian Council of Animal Care. A total of 14 zebrafish (*Danio rerio*) were used to prepare the Ig libraries from 20 clusters and 4 tissue aggregates (**Table S1**). We used 4 unvaccinated ~18 month old zebrafish for the first run. For the second run we vaccinated ten ~18 month old zebrafish intraperitoneally (i.p.) with 2 μg of phycoerythrin (PE; Columbia Biosciences) or bovine serum albumin (BSA) conjugated to Alexa-647 emulsified in complete Freund’s adjuvant (Sigma), followed 1 month later by a second i.p. injection with 2 μg of either BSA or Keyhole Limpet Hemocyanin (KLH) conjugated to Alexa-647 emulsified in incomplete Freund’s adjuvant. Fish were dissected at indicated time points following boosters. The spleen and kidney (a known and a presumed secondary lymphoid tissue) were removed using a dissecting microscope. Then the spleen or kidney was transferred to a microscope slide, and we used fluorescence microscopy to identify the clusters and micro-dissection scalpel was used to lift each MMΦC to another part of the slide to be photographed in brightfield and fluorescence. In addition, we retained the surrounding tissues after removing MMΦCs in the kidney from vaccinated fish to compare the number and clonal relatedness of B-cells between the clusters and their surroundings. Whole kidney and whole intestine were used as positive controls for total V_H_ family expression. Intestine was included as IgT/Z appears to be the predominant isotype of mucosa (22), and we thought that the IgZ repertoire might be broader there than in spleen or kidney.

### Libraries preparation and sequencing

Total RNA was extracted from each cluster (or tissue) using RNeasy Micro Kit (QIAGEN) following the manufacture’s protocol. The concentrations of the total RNA were determined using NanoDrop (ND-1000 spectrophotometer, NanoDrop) and Qubit (RNA HS Assay Kit, Thermo Fisher Scientific). Total RNA from each sample was split into two cDNA synthesis reactions and reverse transcribed using two gene-specific primers. Previously described (23) primers using both IgM and IgZ constant regions were used for cDNA synthesis. Reverse transcription was carried out using SuperScript III First-Strand Synthesis kit (Invitrogen). In a 20 μl reaction cDNA synthesis reaction was carried out at 25°C for 5 minutes, followed by 55°C for 60 minutes, then the reaction was inactivated by heating at 70°C for 15 minutes. Subsequently, RNase H (Invitrogen™) was added to the reactions and incubated at 37°C for 20 minutes to remove complementary RNA.

We used previously described and validated primers (**Table S2**) to capture the variable domain of the zebrafish immunoglobulin heavy chain (23). The forward primers were designed using the consensus leader sequences of the 39 functional V gene segments of the heavy chain, and the reverse primers using the IgM and IgZ constant regions. Initially, these primers were used to verify the expression of IgM and IgZ within zebrafish MMΦCs. Using Phusion™ High-Fidelity DNA Polymerase (Thermo Fisher Scientific), the initial denaturation was carried out at 98°C for 30 seconds, followed by 20 cycles of denaturation at 98°C for 10 seconds, primers annealing to DNA at 60°C for 30 seconds, and extension at 72°C for 30 seconds. This was followed by 10 cycles of denaturation at 98°C for 10 seconds, primers annealing to DNA at 57.1°C for 30 seconds, and extension at 72°C for 30 seconds. The final extension was at 72°C for 7 minutes. After verifying the expression of IgM and IgZ within MMΦCs, multiplex PCR primers were modified by the addition of Illumina forward and reverse adapters to the 5’ of each primer. However, using the modified primers, we were not able to use the 27 forward primers in a single PCR reaction due to primer-dimer formation. Therefore, the 27 forward primers were split into 6 multiplex PCRs, and a total of 12 multiplex PCR reactions were carried out for each sample (for IgM and IgZ).

Multiplex PCR reactions were followed by size selection and cleanup using a 1:1 sample to bead ratio using Ampure XP beads (Beckman Coulter) following the manufacture’s protocol. The concentrations of the purified multiplex PCR products were determined using NanoDrop (ND-1000 spectrophotometer, NanoDrop). Subsequently, a second PCR was done using the purified samples and different combinations of index primers for different samples to allow multiple samples to be sequenced together. Q5^®^ Hot Start High-Fidelity DNA Polymerase (New England Bio Labs nc) was used, and the PCR reaction began with an initial denaturation at 98°C for 3 minutes, followed by 8 cycles of denaturation at 98°C for 30 seconds, annealing of primer to DNA at 55°C for 30 seconds, extension at 72° C for 30 seconds, and a final step at 72° C for 5 minutes.

Then a second cleanup step was carried out using a 1:1 sample to bead ratio using Ampure XP beads (Beckman Coulter) following the manufacture’s protocol. Then, the concentrations of the purified index PCR products were determined using NanoDrop (ND-1000 spectrophotometer,NanoDrop).

To quantify the intact dsDNA in each sample, we used Qubit dsDNA HS (High Sensitivity) kit (Thermo Fisher Scientific, Q32854). Then based on the different concentrations obtained using the Qubit, samples were diluted to pool the exact concentration of individual samples for each library. Afterward, to confirm the size range and check the quality and purity of the prepared libraries, we used the bioanalyzer high sensitivity DNA kit (Agilent Technologies, 5067–4626) following the manufacture’s protocol. Then we used two MiSeq Reagent Kit v2 (500-cycles) MS-102-2003 2x250 (Illumina) to sequence the two Ig repertoire libraries (prepared from unvaccinated and vaccinated zebrafish) using 30% PhiX spike-in.

### Sequence data quality filtering and read assembly

Around 700,000 raw, unassembled reads were obtained per cluster. Remnant Illumina adapters were removed from the sequencing reads using Cutadapt (24), using forward and reverse adapter sequences. Quality filtering and read assembly were performed using several subcommands from pRESTO software package (25). We used align subcommand of MaskPrimers to remove the V- and C-region primers using cut mode. Raw reads that failed primer identification by Maskprimers were removed. Then PairSeq was used to organize and match sequence records with matching coordinates in the files of the forward and reverse reads of each sample. Subsequently, we used align subcommand of AssemblePairs to merge the overlapping paired-end reads into a single sequence by aligning the ends (25).

To identify and remove low-quality reads, we used quality subcommand of FilterSeq (25), and reads with mean Phred quality scores less than 30 were removed. In addition, reads were filtered by length using length subcommand of FilterSeq. Then, to remove duplicate sequences, we used collapseSeq to collapse identical sequences without allowing any missing nucleotides while collapsing sequences (25). After removing duplicate sequences, we used splitseq command to keep only reads with at least two copies; singletons or reads that were observed only once were discarded (25).

### Alignment and clonal clustering

Following quality filtering and reads assembly, VDJ annotation was performed using IMGT/High V-QUEST using zebrafish IgH locus germline sequences (26). Then we used Change-O package (27), which includes multiple tools to process the annotated Ig sequences using the output of IMGT/High V-QUEST (26). Initially, MakeDb was used to store sequence alignment information in a tab-delimited database file. Then we used distToNearest to determine the clustering threshold for each Ig repertoire to group sequences into clusters which will be used to assign Ig sequences into clones. To determine the clustering threshold, we used nucleotide Hamming distance (ham model) and distToNearest (27). Hamming distance is calculated based on the number of nucleotides at which two sequences of the same length differ (27). distToNearest command provides a bimodal distribution of the sequences by calculating the distance between every sequence and its closest neighbor (27). The resulting histogram provides information about the clonal relatedness of the sequences within each repertoire; clonally related sequences are separated from unrelated sequences (27). By manually inspecting the bimodal distribution histogram, we determined the clustering threshold by using the value that separates the clonally related sequences in the repertoire from the clonally unrelated.

Using the clustering threshold value determined for each Ig repertoire in the previous step, we used DefineClones to assign Ig sequences into clones, using ham model and single-linkage hierarchical clustering (27). Initially, sequences are grouped based on the V gene, J gene, and junction length, then Hamming distance and single-linkage hierarchical clustering are used to assign Ig sequences into clones, where each clone is the offspring of a B-cell responding to an antigen with mutated Ig sequences (27). Hamming distance was normalized by the length in which Hamming distance is divided by the length of the junction. To reconstruct the germline sequence for each clone group, we used CreateGermlines command which reconstructs the germline sequence using the initial alignment data and IMGT-gapped zebrafish germline sequences which we added to the Immcantation framework (26, 27).

### Diversity and sample coverage

Using the total number of unique Ig sequences, the number of observed clones, and their relative abundance (frequency) within each repertoire, we estimated sample coverage, as measured by sample completeness or the proportion of the total number of Ig sequences in a repertoire that belong to the sequences represented in the sample. Using assembled sequencing reads then, the subcommand countClones in the alakazam package was used to determine the total number of sequences and clones in each clonal group and their copy number (28). Subsequently the R package iNext (iNterpolation/EXTrapolation) was used to estimate sample coverage (a measure of sample completeness) for IgM and IgZ repertoires (29). Using species richness (q=0) and sample abundance with a 95% confidence interval, iNext calculates sample coverage using the information provided by countClones (the number of observed sequences, clones, and their frequencies).

To estimate clonal diversity, we used the generalized diversity index (Hill numbers) and the output of CreateGermlines (27, 28, 30). Using alphaDiversity subcommands in the Alakazam package (28), Ig repertoires diversity was calculated using a 95% confidence interval, at diversity orders (q values) from 0 to 8. The total number of clones in a sample (species richness) is calculated at q = 0; the exponential of Shannon’s entropy index (q = 1) considers clones in proportion to their frequency. The inverse of Simpson’s concentration index (q = 2) considers clones in proportion to their frequency and ignores rare clones; thus, it represents the dominant clones, and q = ∞ is the reciprocal of the proportional richness (abundance) of the largest clones (31). As the diversity order (q value) increases, larger clones weigh more (29, 31). To compare multiple Ig repertoires, we used repeated resampling in which samples are standardized by sample completeness (30).

To calculate the complete clonal relative abundance, we used estimateAbundance subcommand in Alakazam and the output of CreateGermlines (27, 28). We used a 95% confidence interval and repeated resampling to resample each Ig repertoire to the same completeness and correct for the variability in total sequence counts between Ig repertoires (27). To visualize the estimated complete clonal relative abundance, we used plotAbundanceCurve subcommands in Alakazam (28).

### Building lineage trees and quantification of selection pressure

Using the output of CreateGermlines, and repertoires with at least a hundred unique Ig sequencing reads, we used IgPhyML (HLP19 model) to build phylogenetic trees and to assess the evolutionary hypotheses of B-cell affinity maturation (27, 32). IgPhyML is a repertoire-wide phylogenetic framework that uses a maximum likelihood (ML) model to estimate tree topologies, branch lengths, and to determine the ratio of replacement (nonsynonymous) to silent (synonymous) mutations (dN/dS) within the CDRs and FWRs. However, IgPhyML estimates are inaccurate for small lineages (32), and therefore, we only used Ig repertoires with at least a hundred unique Ig sequencing reads (the majority of the lineages in the Ig repertoires with less than a hundred unique sequences are small lineages). To visualize IgPhyML phylogenetic trees, we used the Alakazam subcommand readIgphyml, which reads the output of IgPhyML and provides the input for igraph package to plot the lineage trees (28). To visualize the selection pressure values for CDRs and FWRs, we used readIgphyml subcommand (28). Then to combine the selection parameters of different Ig repertoires into a single data frame, combineIgphyml, another subcommand in the Alakazam package (28), was utilized, and to create the heat maps, we used ggplot2 package (33).

Using the output of splitseq and sequences with at least two copies, we used B-cell repertoire inductive lineage and immunosequence annotator (BRILIA 3.5.7) software which simultaneously annotates genes, clusters sequences, identifies SHM, and assembles lineage trees based on CDR3 sequence, length, and VJ gene family (32). Using BRILIA VDJ annotation is done by maximizing the total alignment score, which is calculated based on the number of matched and mismatched nucleotides between the Ig repertoire sequences and the germline sequence (32). BRILIA uses the percent of Hamming distance to construct lineage trees (32).

### Antigen trapping by cells within MMΦCs

Because zebrafish have a relatively small number of leukocytes, we used another cyprinid, goldfish (*Carassius auratus)* as a proxy for some antigen trapping assays.

Goldfish (+30 g) and ~18 month old zebrafish were vaccinated with 50 μg or 2 μg (respectively) KLH or BSA conjugated to Alexa-647 (Invitrogen), emulsified in complete Freund’s adjuvant (Sigma). One month following the injection, fish were dissected, and the spleen and kidney were removed and identified. A Zeiss Axio Imager M2 fluorescent microscope was used to assess where in the organs Ag was retained, and also to isolate MMΦCs from organs. Additional, similarly vaccinated fish were sacrificed 14 days post-injection, at which point we’d anticipate any cell that had captured the antigen for peptide presentation, would have degraded the antigen. Spleen and kidney tissues were dispersed sequentially in 500 μm and 40 μm cell strainers prior to layering on 51% percoll to separate leukocytes from erythrocytes (spleen) or glomeruli (kidney). Leukocytes were then fixed in 2% paraformaldehyde. Leukocytes were then assessed two ways: 1) cytospin slides were prepared for confocal fluorescence imaging to see which cell type(s) had bound Ag. 2) Goldfish leukocytes were subjected to FACS analysis with a FACSAria (BD Biosciences) flow cytometer. Unvaccinated goldfish peripheral blood leukocytes were used as non-fluorescing controls to set gates as previously described (18).

### Intact Ag trapping on the surface of MMΦs

Another pair of goldfish were vaccinated as above with just BSA. Isolated leukocytes were incubated with Protein G magnetic beads (Dynabeads; Thermofisher) bound with one of: Rabbit polyclonal anti-BSA (Thermofisher), Rabbit polyclonal anti-mouse IgG (control; Thermofisher), or a no Ab control. Cells were washed and eluted as per the manufacturer’s protocol, and then imaged with a fluorescence microscope.

### Analysis of MMΦC cell numbers

A total of 16 clusters from the spleen and 15 clusters from the kidney were used in two separate imaging flow cytometry runs. Individual clusters were photographed in brightfield and fluorescence before being processed. Clusters were mashed through a cell strainer as above and then the nuclei of the dissociated cells were stained using Hoechst 33342 fluorescent stain (Thermo Fisher Scientific). The total number of cells was assessed using manual cell counts (hemocytometer) before analyzing the samples through an imaging flow cytometer (ImageStream). Data were analyzed using IDEAS 6.1.822.0 software.

## Results

### Evidence for B-cell clonal expansion and mutagenesis within MMΦCs

We hypothesized that if MMΦCs were functioning in a manner analogous to GCs, this could be demonstrated by tracking mutations in the VDJ exons of clonally expanding B-cell clones within isolated clusters.

To test our hypothesis, we isolated auto-fluorescing MMΦCs from the spleen and kidney of zebrafish, using a fluorescence microscope. The kidney is a hypothesized (34), but as yet unproven, secondary lymphoid organ of fishes. Using previously described and validated multiplex PCR primers to amplify all expressed μ- and ζ-chain transcripts from the leader sequence to the first constant domain (23), we prepared and sequenced 24 VDJ repertoire libraries (**Table S1**). For one set of libraries, we used 4 unvaccinated zebrafish, and for another set we used 10 fish vaccinated with KLH, PE, and/or BSA conjugated to Alexa-647. As positive controls for total V_H_ family expression, we screened immunoglobulin repertoires prepared using the whole kidney, intestine, and tissues surrounding MMΦCs in the kidney (**Table S1**).

To calculate the species richness of VDJ recombination clones and sequence coverage from each cluster starting from ~100,000 bidirectionally sequenced and assembled reads per library, we used iNEXT (R package iNterpolation and EXTrapolation). Sequencing coverage was at least 93% for both isotypes in clusters (**Table S1**). The coverage for the whole kidney was 85% for IgM and 98% for IgZ, and the intestine had 96% and 99% coverage for IgM and IgZ isotypes, respectively (**Table S1**). All known Ig V_H_-elements were represented within the VDJ repertoires of these two whole tissues (**Table S3**). In two kidney’s, from which MMΦCs had been removed (i.e., surrounding tissues), there were no IgZ sequences and the coverage for IgM was 97% (**Table S1**). The whole kidney, and a whole intestine had 5539 and 3823 total unique VDJ reads, respectively. VDJ repertoires of individual clusters of unvaccinated fish contained between 1064 and 2725 total unique sequencing reads, and clusters from vaccinated fish had between 510 and 3660 unique sequencing reads (**Table S1**). To be considered ‘unique’ a VDJ sequence had to be found twice in a sequencing pool for an individual cluster or tissue.

To determine the number of cells in a cluster we isolated, pooled, and then dispersed multiple clusters and enumerated total cell counts using either a hemocytometer or electronically using ImageStream. Imagestream analyses of cells from MMΦCs isolated from the spleen (n = 16 clusters) and kidney (n = 15 clusters) identified, on average 33,252 cells/cluster and 37,091 cells/cluster, respectively. Using the ImageStream IDEAS software, we selected the cells in focus and separated them from debris, doublets, and aggregates. Based on the cell size and internal complexity we identified 5305 lymphocyte-like cells in individual clusters (n = 16) isolated from the spleen and 6027 cells/cluster (n = 15) isolated from the kidney. Similar cell numbers were obtained by hemocytometer counts. These lymphocyte-like cell numbers were consistent with the number of unique clones identified in clusters above, allowing for some unmutated daughter cells.

**Figure 1:**
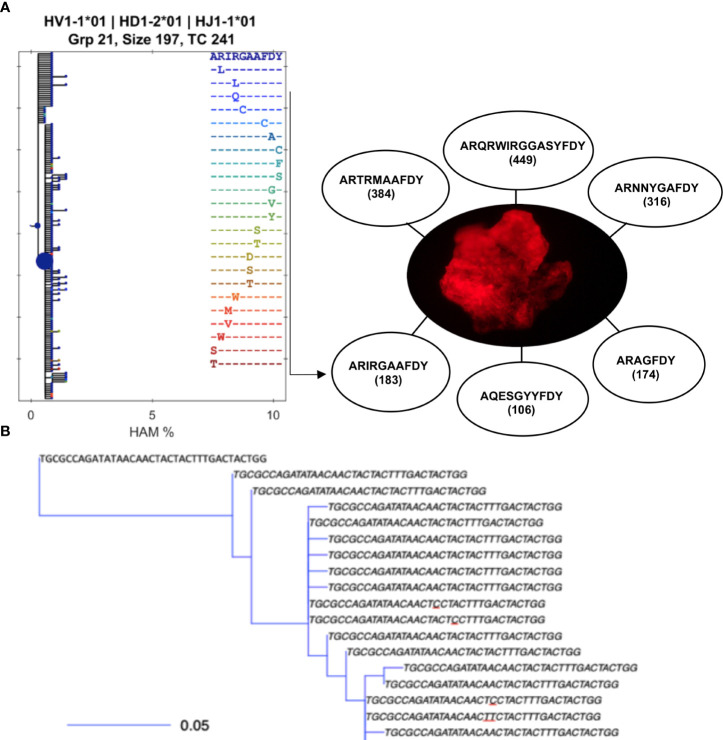
B-cell clonotypes proliferate within MMΦCs while acquiring mutations in the variable region of Ig genes. **(A)** Translated sequences of the CDR3 of the dominant clones within a single MMΦC (F4UKCa) and the number of putative daughter cells. Lineage tree using BRILIA shows only the different CDR3 sequences (there are 183 unique sequences in this clone but only 24 unique CDR3 sequences). Each dot of the left hand tree represents a unique sequence, and each dot color corresponds to a unique CDR3 sequence. The dot size reflects the total copy number of each sequence. The x-axis shows the percent of Hamming distance calculated based on the number of nucleotides at which two sequences of the same length differ. **(B)** An example of part of a smaller lineage tree using BuildTrees, IgPhyML (HLP19 model) and Alakazam (igraph), CDR3 nucleotide sequences of all the unique sequences (the size of this sub-clone is 35 sequences). A few of the CDR3 sequences are unique to specifc sequences with mutated nucleotides (underlined nucleotides). Some branches have their defining mutation outside of the CDR. The scale bar represents the branch length (expected number of substitutions per codon site). The analysis was done using IgPhyML and BRILIA. HAM %, ​is the percent of Hamming distance which is calculated based on the number of nucleotides at which two sequences of the same length differ.

To determine the number of B-cell clonotypes within a cluster, we constructed Ig clonal lineage trees using a B-cell phylogenetic inference package (35). This revealed that while the size of the clones varies among the different clusters, generally, it appears that each cluster is dominated by a few clonally expanding B-cell clonotypes (as inferred from VDJ sequences), with some of the dominant clones having more than 400 uniquely mutated daughter cells as assessed using B cell phylogenetic inference package (28, 35) (**Tables S3, S4** and **Figures 1A, B**). Similarly, using BRILIA software to assemble clonal lineages (32), in each cluster we found a few clonally expanding clonotypes, with some of the dominant clones having up to 300 daughter cells (**Figure 1A**). Clones smaller than dominant clones, were also found within MMΦCs (**Tables S4 and S5**, e.g., **Figure 1B**). One interpretation of clones of varying sizes is that MMΦCs have ongoing plasmablast recruitment, and clusters develop toward the dominance of a few clones and their progeny. In MMΦCs from vaccinated fish the clusters had mainly IgM^+^ B-cell clonotypes with some clusters having fewer than a hundred or no IgZ^+^ B-cell clonotypes. In contrast several MMΦCs from unvaccinated fish had IgZ as the predominant isotype (**Tables S1, S4, S5**). Unvaccinated fish are still exposed to environmental antigens, such as gut microbiota. This indicates that the role of MMΦCs in the development of Ag-specific B-cells is not limited to IgM^+^ B-cells but also involves IgZ^+^ (or IgT^+^) B-cells, which has been described as the primary antibody isotype in mucosal immunity in teleosts (22). Kidney tissues surrounding the clusters had five times fewer (on average 105 reads) total unique B-cells than the clusters, which would be consistent with MMΦCs being the sites where B-cells proliferate and acquire mutations before migrating to the surrounding tissues. Several VDJ sequences with no mutations were found associated with isolated clusters. These clones could be from naive B-cells, which is consistent with the presence of naive follicular B-cells within (or surrounding) ongoing GCs (36, 37). Our data show that each cluster has a few dominant clonotypes, and some smaller clones.

**Figure 2:**
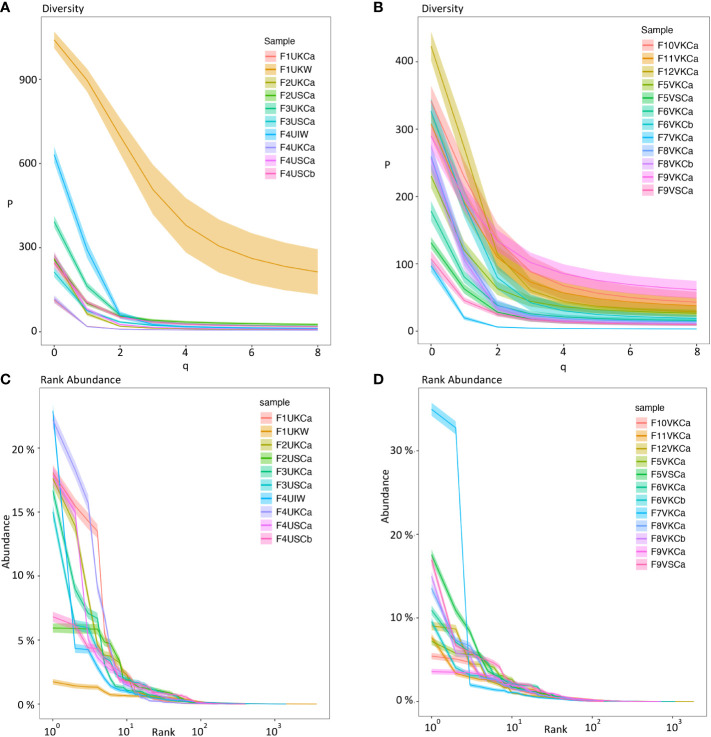
Diversity index (*^q^D*) (Hill numbers) **(A, B)** and clonal (rank) abundance curves **(C, D)** using Ig sequences from MMΦCs and whole kidney and intestine. **(A, C)** MMΦCs from unvaccinated zebrafish and the whole kidney and intestine. **(B, D)** MMΦCs from vaccinated zebrafish. ^q^D is the diversity number, q values are the order of the diversity number. q = 0 is the total number of species in a sample, q = 1 is the exponential of Shannon’s entropy index (considers species in proportion to their frequency), and q = 2 is the inverse of Simpson’s concentration index (considers species in proportion to their frequency and ignores rare species). q=∞ is the reciprocal of the proportional richness of the commonest species. Rank abundance is the Ig clone size as a percent of the repertoire. The shaded areas are 95% confidence intervals. The analysis was performed using Alakazam. Sample designations are (fish number, vaccination status, & tissue source): F# - Fish #; U/V - Unvaccinated/Vaccinated; cluster from K/S - Kidney/Spleen and cluster # - a/b; or W -whole tissue; I - intestine.

To visualize the clonal diversity for each repertoire, we used Hill numbers where higher orders (q values) give more weight to larger clones. Using repeated resampling to correct for the variability in sequencing depth between samples, we found low clonal diversity and more related clones within repertoires from individual MMΦCs from unvaccinated (**Figure 2A**) and vaccinated fish (**Figure 2B**). All the repertoires from MMΦCs had a low clonal diversity number compared with the whole kidney. At q=2 (the inverse of Simpson’s concentration index) the dominant clones are represented in proportion to their frequency and rare clones are ignored. Repertoires from MMΦCs had a clonal diversity number less than 150 (at q=2), which is much lower than the diversity number (≈ 650) for the whole kidney (F1UKW; **Figure 2A**) at the same order. We also included one sample of whole intestine (F4UIW), and from our analysis we can infer that clonal expansion of B-cells and loss of clonal diversity in the intestine is comparable to that detected in MMΦCs, which suggests that affinity modification occurs somewhere in the intestine (F4UIW; **Figure 2A**). All clusters appear unique in number of clonotypes, with all having some dominant clonotypes. In vaccinated fish we see evidence for more clonotypes in some clusters (F9VSCa) compared to others (F7VKCa) (**Figure 2B**).

To determine the Ig clone size as a percent of the total repertoire, we calculated the clonal abundance distribution for individual clusters using Alakazam. We found high clonal abundance and low diversity within MMΦCs from unvaccinated or vaccinated fish (**Figures 2C, D**). In whole kidney no individual clonotypes appear to dominate, while the intestine shows a different profile with a few dominant clonotypes (**Figure 2C**). In unvaccinated fish, the cluster F4UKCa shows the lowest clonotype diversity. This cluster has 6 major clonotypes and the clonal expansion is shown in **Figure 1A**. In vaccinated fish, the cluster F7VKCa had the lowest clonotype diversity. In this sample, two clonotypes account for 42.7% of the cluster Ig repertoire (**Figure 2D** and **Table S5**). Taken together our data is consistent with the clonal expansion of a limited number of B cells within each cluster.

### Evidence for B-cell selection within MMΦCs

**Figure 3:**
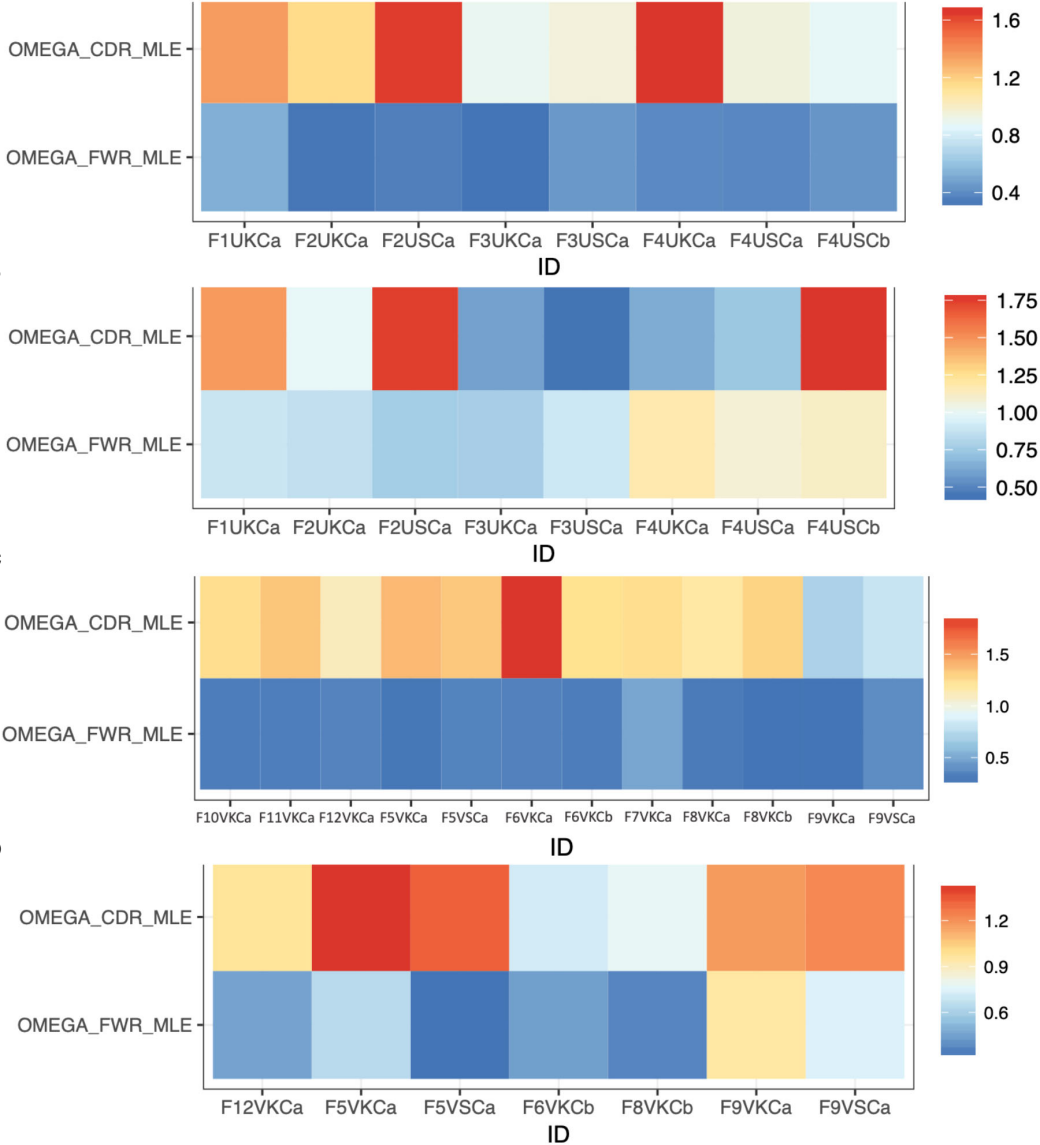
Selection estimates (the ratio of replacement to silent mutations, R/S) for CDRs (^*ω*^CDR) and FWRs (^*ω*^FWR) using Ig repertoires isolated from MMΦCs from zebrafish. **(A)** Unvaccinated (IgM isotype). **(B)** Unvaccinated (IgZ isotype). **(C)** Vaccinated (IgM isotype). **(D)** Vaccinated (IgZ isotype). Refer to **Table S1** for detailed descriptions of the samples used. The analysis was performed using IgPhyML HLP19 model.

To determine if there is an active Ag-driven selection process in MMΦCs, we used the Ig VDJ repertoires generated from each cluster to examine the ratio of replacement to silent mutations (R/S) in the complementarity determining regions (CDRs) and the framework regions (FWRs) of the VDJ exons. CDRs are the sites that determine the Ag-binding affinity and specificity, while FWRs provide the structural backbone of the Ig. Although R/S ratio varied among the different repertoires, their CDRs typically had a higher R/S estimate compared to the FWRs (**Figures 3A–D**). In an unvaccinated fish, the cluster with the lowest clonotype diversity (F4UKCa), shows the highest selection in the CDR region of IgM (**Figure 3A**), although the opposite is true for IgZ and four of the top five clonotypes are IgZ (**Figure 3B** and **Table S4**). In vaccinated fish, all clusters show evidence of selection in the CDR in IgM (**Figure 3C**), and IgZ (**Figure 3D**). The R/S ratio in the CDR differs for each cluster (even if from the same kidney F6VKCa or b), and each cluster represents several clonotypes, only some of which may reveal evidence of selection. These results are consistent with an active Ag-driven selection process within MMΦCs.

Some of the repertoires for IgZ isotype from MMΦCs from unvaccinated zebrafish had a lower R/S ratio in the CDRs and higher R/S ratio in the FWRs (**Figure 3B**), which is a sign of negative selection. Interestingly, these repertoires had an average mean tree length of 0.65 (i.e., the average expected substitutions per codon site). This mean tree length is long compared to IgM isotype repertoires from MMΦCs from vaccinated and unvaccinated fish; average mean tree length of 0.13. One interpretation of a longer tree length is that the cells had been acquiring mutations for a longer time, perhaps by cycling back to the centroblast stage. We found a positive correlation between the mean tree length and the R/S ratio in the FWRs and all the IgZ repertoires with longer mean tree length had a high R/S ratio in their FWRs (**Figures 3B, D** and **Figure S2**). IgM repertoires had a R/S ratio in their FWRs between 0.28-0.5; however, this ratio reached 1.17 in the IgZ repertoires (**Figure S2**). Together, these results indicate that the negative selection found in IgZ isotype (High R/S ratio in the FWRs) suggests that perhaps the binding affinity of these immunoglobulins reached an optimal affinity, and the addition of mutations is not beneficial and possibly selected against. Similar observations were found in response to influenza vaccine and HIV broadly neutralizing antibodies (38, 39). Kleinstein and colleagues also imagined that the Ab lineages to these viruses had accumulated (positively selected for) mutations in their CDRs until reaching an affinity peak, after which further CDR mutations would become deleterious, and selected against, whilst mutations in the FWRs that were not deleterious would accumulate, and they suggested that eventually shifting to negative selection is a general feature of prolonged or repeated affinity maturation (38). In our case we are imagining that it is gut microflora that is providing the protracted stimulation of IgZ lineages, in the absence of competing (i.e. vaccine) stimulation in the unvaccinated fish.

### Ag is trapped within MMΦCs by melano-macrophages

**Figure 4:**
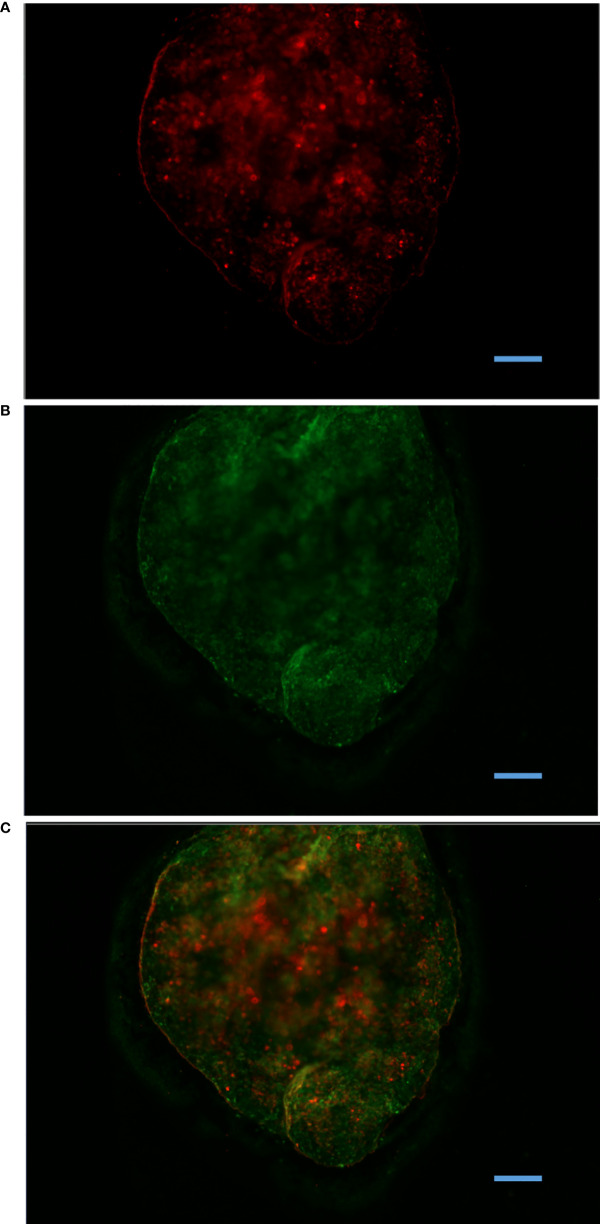
Antigen retention within MMΦC isolated from zebrafish spleen. Spleen MMΦC isolated from zebrafish immunized with BSA-Alexa647, one month following the vaccination. **(A)** BSA-Alexa-647 is visible in the Cy5 channel. **(B)** Auto-fluorescent MMΦs are visible in the FITC channel. **(C)** Merge. Alexa647 was only observed in clusters, and not in surrounding tissues.

**Figure 5:**
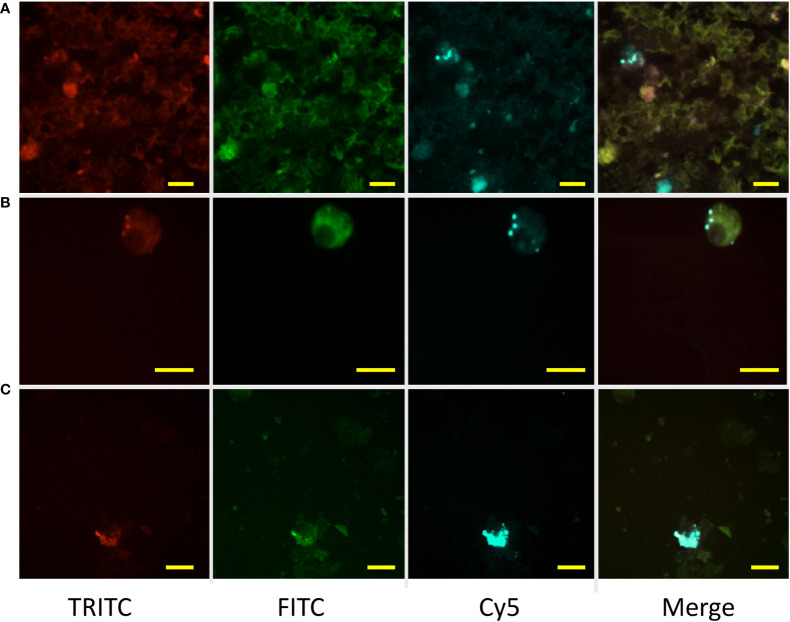
Retention of experimentally injected labeled antigen by MMΦs. Leukocytes were isolated from goldfish or zebrafish spleen and kidney 14 days after immunization with BSA-Alexa-647. Red (TRITC) and green (FITC) are MMΦ auto-fluorescence, while BSA-Alexa 647 is seen in the Cy5 channel. Distinct blue spots in the merged image indicate the signal from Alexa-fluor 647. **(A, B)** Kidney and spleen leukocytes isolated from immunized goldfish. **(C)** Kidney leukocytes isolated from zebrafish immunized with Alexa-647. Scale bars are 10 µm.

**Figure 6:**
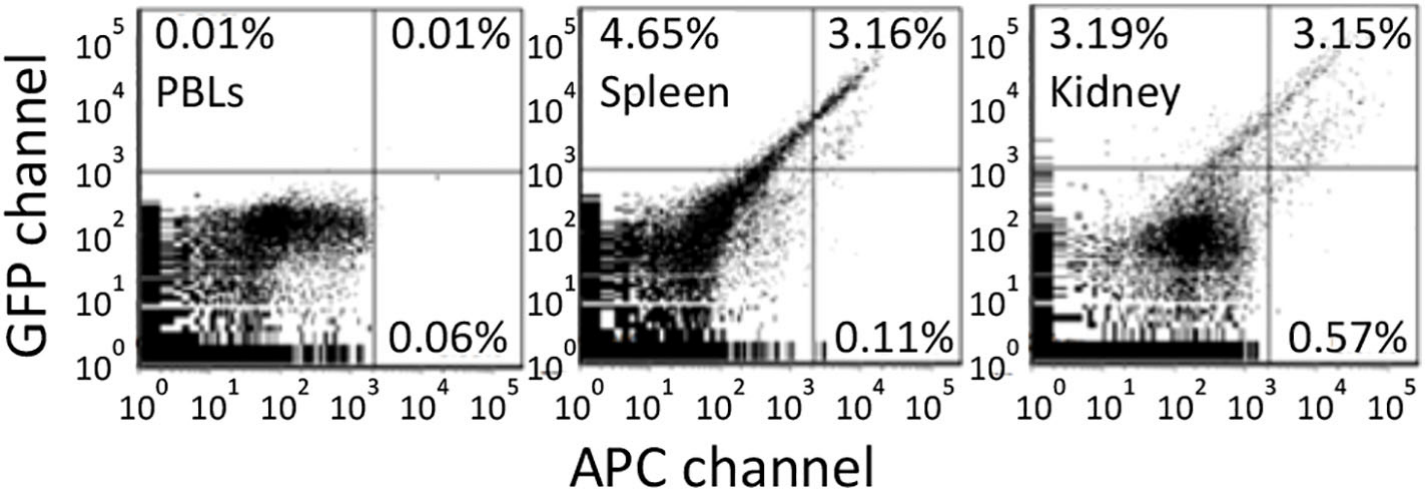
Antigen retention of far red tagged keyhole limpet hemocyanin (KLH) by melano-macrophages from immunized goldfish. Goldfish were immunized with KLH and boosted once over a year. These goldfish were then injected with Alexa Fluor 647 tagged KLH and FACS analyzed 13 days later. Green fluorescence is in the GFP channel while far red is done in the APC channel. Background fluorescence was determined using peripheral blood leukocytes. Spleen and Kidney leukocytes contained approximately 3% double positive and <1% single far red positive cells.

Prior studies in fish had established that long-term Ag retention occurred at, or adjacent to MMΦs (19, 20). Here we verified this in goldfish and zebrafish MMΦ clusters (**Figure 4** and not shown) and MMΦ cells (**Figure 5**). To visualize the location of the antigen in the MMΦ cells we used dynamic (Z-stack) confocal imaging, which suggested that the antigen was both within the cell and on the cell surface (**Figure 5**). To establish whether Ag was retained in an intact form on the cell surface, fixed spleen and kidney leukocytes from unvaccinated goldfish or fish vaccinated with BSA, were exposed to magnetic beads conjugated to anti-BSA polyclonal antibodies, control PAb or a no Ab control. Auto-fluorescent melano-macrophages only bound to beads conjugated with the anti-BSA PAb, and this was only if they came from a fish vaccinated with BSA (**Figure S3**). A small number of non-autofluorescent cells bound to the beads regardless of the Ab bound to them. To ensure that only melano-macrophages were retaining Ag, goldfish previously vaccinated and boosted with KLH, were given an i.p. injection of KLH-Alexa-647. After 2 weeks spleen and kidney leukocytes were FACS analyzed for melano-macrophages (green auto-fluorescence) and far red (KLH-Alexa647 Ag; **Figure 6**). Thirty to 50 percent of the melano-macrophages were also far red positive. Less than 1% of events were Alexa-647 positive, and negative in the green fluorescence channel (**Figure 6**). The source of MMΦ auto-fluorescent pigments, lipofuscin and hemosiderin, are believed to be from the uptake and breakdown of effete RBCs and apoptotic cells (40). Melano-macrophages accumulate pigments over time (41, 42) and immature cells may have too little pigment to be detected by FACS (cf. **Figure S3**). These GFP negative, Alexa-647 positive cells could also be B-cells with Alexa-647 specific Abs. Similar FACS done on leukocytes from BSA-Alexa647 had less than 10% Alexa positive melano-macrophages (not shown), though this was not unexpected as BSA has less epitope diversity, and likely immunogenicity than KLH. Our data support the hypothesis that melano-macrophages are capable of trapping intact antigen on their surface, suggesting they are performing the role of follicular dendritic cells from mammalian germinal centers.

To investigate whether FDC markers could be identified in MMΦs we used RT-PCR and mined a transcriptome of goldfish kidney MMΦs. FDC markers such as BAFF, FcR-like, CR1-like, and MFGE8 were found to be expressed in goldfish kidney MMΦs (**Table S6**). Several pIgR-like gene transcripts were also identified from MMΦs. Parallel analyses on cDNA from reticular cells, which envelope each cluster, failed to find these markers, though a positive control marker (β-actin) was amplified. These findings are consistent with melano-macrophages playing a role in selection and survival of antigen binding centrocytes, and elimination of apoptotic centrocytes in the case of MFGE8.

## Discussion

Here we show that fish melano-macrophage clusters are sites of antibody affinity modification and selection analogous to mammalian germinal centers. The existence of a germinal center analogue in early vertebrates is not unexpected, as unregulated Aicda expression is known to be highly oncogenic, and somatic hypermutation without subsequent cell selection is associated with propagation of autoimmune responses. Thus, there would be strong selective advantages to organisms that could regulate and contain hypermutated cells within a discrete microenvironment such as a germinal center analogue.

Virtually all teleost fish have melano-macrophage clusters; these clusters have been associated with antigen trapping (19–21) and immune responses, as they have been observed to increase in size following vaccination or infection (43–45). Further, they have also been shown to have cells expressing CD4, a T_H_-cell marker (16). In this study, VDJ repertoire analyses reveal hallmarks of germinal center responses: 1) clonal expansion of B-cell (centroblast) clones with concomitant somatic hypermutation of VDJ exons. 2) Replacement/Silent mutation patterns consistent with positive selection of centrocytes. 3) A selection platform, in the form of melano-macrophages which retain intact antigen on their surface. The melano-macrophages also express several genes which are putative orthologues of those used by FDCs to display intact Ag complexes for selection of centrocytes, or which facilitate removal of unselected, apoptotic cells (MFGE8).

Overall, these findings suggest that melano-macrophage clusters are functionally analogous to homeotherm germinal centers. They also indicate that the kidney of bony fishes is a *bona fide* secondary lymphoid tissue.

While we have not herein demonstrated affinity maturation at the protein level, it has been quite convincingly demonstrated in two other teleost fish. Ye and colleagues used antibody affinity partitioning to demonstrate shifts in affinity sub-populations following vaccination of rainbow trout and channel catfish (11, 12). Most striking was that in trout, several high affinity sub-populations that were not present 5 weeks after immunization, emerged thereafter. This is inconsistent with the argument that increases in higher affinity antibodies might only be the result of activation of B-cells that already had high affinity antibodies, and that there is not affinity maturation. Aside from our observations of somatic hypermutations there are studies from sharks, fish and amphibians, that Ig V(D)J exons acquire *de novo* mutations not seen in the germline V- and J-elements [reviewed in (46)]. Perhaps most intriguing among these is of the shark Ig_NAR_ isotype which is encoded entirely by a single heavy chain. Flajnik and colleagues (47) immunized nurse sharks with HEL and then made phage display libraries so they could analyze anti-HEL Ig_NAR_ antibodies and genes. They noted distinct clones that seemed to have acquired successive mutations. Concomitant with increased mutation, was increased HEL binding affinity. What hadn’t been established until now, is the cellular context in which hypermutation, and presumed selection of affinity matured B-cells occurred.

It appears that the selection platform for affinity modified B-cells has also changed over time. Various ultrastructural studies of fish lymphoid tissues have sought to identify with the morphology of follicular dendritic cells, without success. An attempt to identify fish FDCs using CAN-42, a MAb against a mammalian FDC carbohydrate Ag, only stained fish melano-macrophages (48). In goldfish and zebrafish it appears that melano-macrophages are performing some of the roles associated with FDCs, including cell surface display of intact Ag, as well as the probable production of BAFF and MFGE8.

In the amphibian *Xenopus* the Ag display roles would appear to be performed by ‘XL’ cells which have the morphology and phenotypic characteristic of dendritic cells (49). Like melano-macrophages, the XL cells produce various FDC-associated molecules, such as the B- and T-cell chemo-attractants CXCL13 and CCL19 (respectively), the apoptosis rescue cytokine BAFF, as well as an IgM receptor pIgR2 (49).

The next step of our work will be to do single cell transcriptome sequencing to get an idea of the proportions and state of different cell types in the isolated melano-macrophage clusters. Key among these will the proportion of melano-macrophages and (follicular)? T_H_ cells both of which we believe will be key to B-cell selection. We predict that the cellular environment of the clusters be such that in cases where there is an excess of antigen (e.g. following vaccination or systemic infection) there will be selection of most B-cells with antibodies that can bind the antigen/pathogen. We would see this as advantageous to small animals, such as zebrafish, which necessarily have a relatively small number of circulating naïve, or memory, B-cells to respond to an infection.

Our findings will help develop comprehensive models of how antibody affinity maturation functions in fish. The long-term application of this work is in improving the efficacy of vaccines used in the aquaculture and fish husbandry industries. These findings may also be of interest to those studying antibody-based autoimmunity where GCs form in the absence of FDCs in lymphoid tissues or in the inflamed tissue like in the synovial tissue in patients with rheumatoid and reactive arthritis (50, 51).

## Data availability statement

The datasets presented in this study can be found in online repositories. The names of the repository/repositories and accession number(s) can be found in the article/**Supplementary Material**. Ig repertoires and RNA-seq data were deposited in the NCBI SRA repository under accession numbers PRJNA851813, PRJNA852545, and PRJNA852554.

## Ethics statement

The animal study was reviewed and approved by University of Alberta Animal Care and Use Committee.

## Author contributions

BGM and DW designed experiments. DW, AM, C-WF and HA conducted experiments. DW did all computation. All authors contributed to the article and approved the submitted version.
