# Supplementary material for: Immunoglobulin VDJ repertoires reveal hallmarks of germinal centers in unique cell clusters isolated from zebrafish (Danio rerio) lymphoid tissues
Source: Front Immunol. 2022 Dec 8;13:1058877. doi: 10.3389/fimmu.2022.1058877 (PMC9772432; doi:10.3389/fimmu.2022.1058877)
Supplement: Supplementary file 1 [file DataSheet_1.pdf]

Supplemental Tables and Figures for FImm 1058877 Waly et al., zebrafish germinal center analogues.

`Table S1. Ig repertoires isolated from MMΦCs or tissues from unvaccinated and vaccinated fish VDJ combination coverage, number of unique reads, and the total number of clones for IgM and IgZ isotypes. VDJ repertoires are available at NCBI SRA PRJNA851813 and PRJNA852545.

Fish[1]	Vaccination[2]	IgM isotype			IgZ isotype		
		Coverage %	Number of unique reads[3]	Total number of Clones	Coverage %	Number of unique reads[3]	Total number of Clones
F1UKCa	none	98.8	1052	154	99.1	878	10
F2UKCa	none	97.4	1495	366	99.6	1166	24
F2USCa	none	98.4	926	256	99.1	138	7
F3UKCa	none	93.5	876	437	99.2	336	21
F3USCa	none	99	2307	332	99.5	418	9
F4UKCa	none	98.6	989	169	99.6	1335	10
F4USCa	none	98.5	1601	360	99.5	406	11
F4USCb	none	98.6	1898	358	98.9	462	14
F5VSCa	1º PE-Alexa 2º BSA-Alexa	98.5	416	137	95.5	176	84
F5VKCa	1º PE-Alexa 2º BSA-Alexa	98.7	1569	478	98.9	416	31
F6VKCa	1º PE-Alexa 2º BSA-Alexa	98.5	1112	262	-	0	0
F6VKCb	1º PE-Alexa 2º BSA-Alexa	98.3	3295	992	98.3	253	33
F7VKCa	1º PE-Alexa 2º BSA-Alexa	98.1	421	99	96.8	89	2
F8VKCa	1º PE-Alexa 2º BSA-Alexa	98.8	2826	675	96.3	48	13
F8VKCb	1º PE-Alexa 2º BSA-Alexa	98.7	2513	672	98.9	111	22
F9VSCa	1º BSA-Alexa 2º KLH-Alexa	98.5	612	125	98.8	151	8
F9VKCa	1º BSA-Alexa 2º KLH-Alexa	98.5	1862	610	99.4	189	13
F10VKCa	1º BSA-Alexa	98	2898	970	96.1	66	4
F11VKCa	1º KLH	98.4	2109	687	98.4	89	13
F12VKCa	1º KLH	97.5	3411	1626	98.7	249	77
F1UKW	none	85.9	4389	3511	98.2	1150	413
F4UIW	none	96.8	3155	1394	99.6	668	91
F13VKS	1º BSA-Alexa	97.3	82	32	-	0	0
F14VKS	1º BSA-Alexa	97.7	129	35	-	0	0

[1]F# - Fish # in group; U/V - Unvaccinated/Vaccinated; cluster from K/S - Kidney/Spleen and cluster # - a/b; W -whole tissue; I - intestine; S - tissues surrounding the clusters.

[2]1° Primary, 2° Secondary; PE - Phycoerythrin; BSA - Bovine Serum Albumin; KLH - Keyhole Limpit Hemocyanin; Alexa – Alexa-647

[3] Two copies of the same transcript are required to deem a read to be ‘unique’.

Table S2 – Primers used for generating the zebrafish VDJ repertoires (adapted from 24).

Primer	Sequence 5’-3’
**ZF IGHV 4-1 FWD**	TCGTCGGCAGCGTCAGATGTGTATAAGAGACAGTGGTCTCCTCTGCCTTTTGT
**ZF IGHV 4-2 FWD**	TCGTCGGCAGCGTCAGATGTGTATAAGAGACAGAACCATGATCGCCTCATCTC
**ZF IGHV 4-3 FWD**	TCGTCGGCAGCGTCAGATGTGTATAAGAGACAGGATGGCAACAACATCCTGTG
**ZF IGHV 4-4 FWD**	TCGTCGGCAGCGTCAGATGTGTATAAGAGACAGTGCATTTCAGTTCTGCTGCT
**ZF IGHV 4-5 FWD**	TCGTCGGCAGCGTCAGATGTGTATAAGAGACAGACGAATGCAGGAGTCAGACA
**ZF IGHV 4-6 FWD**	TCGTCGGCAGCGTCAGATGTGTATAAGAGACAGTGTTTCAACTGTTCGTGGTCA
**ZF IGHV 4-7 FWD**	TCGTCGGCAGCGTCAGATGTGTATAAGAGACAGTGGAGTTGTGTTGATGATGATT
**ZF IGHV 4-8 FWD**	TCGTCGGCAGCGTCAGATGTGTATAAGAGACAGTTCATATGCACATGGTCAGTCA
**ZF IGHV 4-9 FWD**	TCGTCGGCAGCGTCAGATGTGTATAAGAGACAGTGTGGTGATTGTCTTTCAAGG
**ZF IGHV 4-10 FWD**	TCGTCGGCAGCGTCAGATGTGTATAAGAGACAGTGGAAAAGGAGTCAAAAAGCAT
**ZF IGHV 4-11 FWD**	TCGTCGGCAGCGTCAGATGTGTATAAGAGACAGGCTTTTGTCATGTTTGCTCTCA
**ZF IGHV 4-12 FWD**	TCGTCGGCAGCGTCAGATGTGTATAAGAGACAGGCTTACTGCTGCTCTCATTCAG
**ZF IGHV 4-13 FWD**	TCGTCGGCAGCGTCAGATGTGTATAAGAGACAGTTTCTGCTGCTGTGCTTTAC
**ZF IGHV 4-14 FWD**	TCGTCGGCAGCGTCAGATGTGTATAAGAGACAGCTGCTGTTTTCATTGGCCTTA
**ZF IGHV 4-15 FWD**	TCGTCGGCAGCGTCAGATGTGTATAAGAGACAGGGTTTATACTGTCAAGGCATGG
**ZF IGHV 4-16 FWD**	TCGTCGGCAGCGTCAGATGTGTATAAGAGACAGCAGCCTCAAGATGAAGAATGC
**ZF IGHV 4-17 FWD**	TCGTCGGCAGCGTCAGATGTGTATAAGAGACAGCTAGTGCTGTTTCTGGCAGT
**ZF IGHV 4-18 FWD**	TCGTCGGCAGCGTCAGATGTGTATAAGAGACAGCATGATCACCTCATCTCTCTGC
**ZF IGHV 4-19 FWD**	TCGTCGGCAGCGTCAGATGTGTATAAGAGACAGCATGATTCTGAGCATTTTATCATGT
**ZF IGHV 4-20 FWD**	TCGTCGGCAGCGTCAGATGTGTATAAGAGACAGCAATAATCAACTCACTCCTGCTG
**ZF IGHV 4-21 FWD**	TCGTCGGCAGCGTCAGATGTGTATAAGAGACAGCTGCGTCCAGTGTATATTCCA
**ZF IGHV 4-22 FWD**	TCGTCGGCAGCGTCAGATGTGTATAAGAGACAGTGTATTGACTGTCAGGTTGTGC
**ZF IGHV 4-23 FWD**	TCGTCGGCAGCGTCAGATGTGTATAAGAGACAGTCTTTCTGCAGTTGGCAG
**ZF IGHV 4-24 FWD**	TCGTCGGCAGCGTCAGATGTGTATAAGAGACAGTCTCAAAGTTGTTGGTGTCAGA
**ZF IGHV 4-25 FWD**	TCGTCGGCAGCGTCAGATGTGTATAAGAGACAGCTCTCTAAACAAGTGCAAAGGTC
**ZF IGHV 4-26 FWD**	TCGTCGGCAGCGTCAGATGTGTATAAGAGACAGTGGACCTTAAACTTAACTGTCTG
**ZF IGHV 4-27 FWD**	TCGTCGGCAGCGTCAGATGTGTATAAGAGACAGCCATATGTTTCTGGCATCTCCC
**ZF C-μ IgM REV**	GTCTCGTGGGCTCGGAGATGTGTATAAGAGACAGTGCACTGAGACAAACCGAAG
**ZF C-ζ IgZ REV**	GTCTCGTGGGCTCGGAGATGTGTATAAGAGACAGTCAGAGGCCAGACATCCAAT

Table S3. Frequency of the amplified V_H-elements isolated from whole kidney and intestine.

Gene	F1UKW (IgM)[1]	F1UKW (IgZ)[1]	F4UIW (IgM)[1]	F4UIW (IgZ)[1]
IGHV1-4	0.12345	0.33656	0.16420	0.00194
IGHV14-1	0.08531	0.17940	0.08919	0.00325
IGHV9-1	0.07838	0.00105	0.13633	0.00000
IGHV4-6	0.07267	0.00231	0.04776	0.00107
IGHV5-1	0.05483	0.00041	0.09607	0.00000
IGHV13-2	0.05285	0.19847	0.05654	0.07516
IGHV10-1	0.05275	0.00018	0.03912	0.00000
IGHV1-1	0.05022	0.08513	0.03846	0.85894
IGHV4-1	0.03563	0.00326	0.01914	0.00000
IGHV5-5	0.03491	0.00003	0.05531	0.00000
IGHV8-3	0.03489	0.00046	0.01233	0.00000
IGHV5-4	0.02817	0.00003	0.01442	0.00004
IGHV1-2	0.02592	0.04472	0.01516	0.00420
IGHV4-8	0.02452	0.00072	0.00511	0.00246
IGHV7-1	0.02442	0.02466	0.06393	0.00000
IGHV5-3	0.02327	0.00000	0.01838	0.00000
IGHV9-2	0.01913	0.02717	0.01071	0.01081
IGHV4-2	0.01750	0.00041	0.00738	0.00000
IGHV9-4	0.01698	0.04644	0.01287	0.00851
IGHV2-1	0.01658	0.00000	0.00175	0.00067
IGHV1-3	0.01592	0.00236	0.01475	0.00000
IGHV11-2	0.01531	0.00249	0.01539	0.00000
IGHV4-9	0.01321	0.00174	0.00887	0.00000
IGHV6-2	0.01282	0.00000	0.00636	0.00000
IGHV2-2	0.01148	0.00010	0.00379	0.00000
IGHV4-3	0.01013	0.00264	0.00311	0.03006
IGHV8-1	0.00933	0.00067	0.00525	0.00000
IGHV3-2	0.00688	0.03310	0.01162	0.00234
IGHV5-8	0.00667	0.00000	0.00515	0.00000
IGHV6-1	0.00540	0.00164	0.00233	0.00000
IGHV4-5	0.00492	0.00003	0.00441	0.00000
IGHV5-7	0.00366	0.00046	0.00343	0.00000
IGHV4-7	0.00358	0.00000	0.00188	0.00000
IGHV11-1	0.00274	0.00074	0.00525	0.00000
IGHV9-3	0.00262	0.00162	0.00285	0.00000
IGHV1-5	0.00181	0.00095	0.00081	0.00055
IGHV5-2	0.00075	0.00000	0.00014	0.00000
IGHV8-4	0.00031	0.00003	0.00000	0.00000
IGHV2-3	0.00007	0.00003	0.00000	0.00000
IGHV8-2	0.00000	0.00000	0.00046	0.00000

[1]F# - Fish # in group; U/V - Unvaccinated/Vaccinated; W - whole tissue; K - kidney: I – intestine.

Table S4. Size of the top clones in Ig repertoires isolated using individual MMΦCs from unvaccinated zebrafish.

	F1UKCa[1]	F2UKCa[1]	F2USCa[1]	F3UKCa[1]	F3USCa[1]	F4UKCa[1]	F4USCa[1]	F4USCb[1]
**Clone**	**Number of unique reads**	**Number of unique reads**	**Number of unique reads**	**Number of unique reads**	**Number of unique reads**	**Number of unique reads**	**Number of unique reads**	**Number of unique reads**
**1**	404	416	50	77	320	449	274	189
**2**	301	324	48	74	162	384	170	97
**3**	238	186	48	74	117	316	92	87
**4**	215	90	45	31	97	183	90	81
**5**	115	79	35	29	91	174	67	65
**6**	60	77	30	19	74	106	49	56
**7**	48	69	28	13	70	63	48	55
**8**	42	69	27	13	65	59	44	44
**9**	38	67	24	12	64	34	42	38
**10**	37	49	24	12	46	30	28	37

[1]F# - Fish # in group; U - Unvaccinated; cluster from K/S - Kidney/Spleen and cluster # - a/b. Shaded cells are clones of IgZ isotype.

Table S5. Size of the top clones in Ig repertoires isolated using individual MMΦCs from vaccinated zebrafish.

	F5VSCa[1]	F5VKCa[1]	F6VKCa[1]	F6VKCb[1]	F7VKCa[1]	F8VKCa[1]
**Clone**	**Number of unique reads**	**Number of unique reads**	**Number of unique reads**	**Number of unique reads**	**Number of unique reads**	**Number of unique reads**
**1**	78	83	93	284	168	261
**2**	43	80	88	110	88	169
**3**	31	71	79	64	10	145
**4**	18	70	41	71	9	102
**5**	16	60	41	51	9	61
**6**	15	60	21	37	9	54
7	13	44	20	37	8	50
**8**	13	42	19	36	7	50
**9**	11	41	18	35	7	33
**10**	10	31	17	33	6	30
	**F8VKCb[1]**	**F9VSCa[1]**	**F9VKCa[1]**	**F10VKCa[1]**	**F11VKCa[1]**	**F12VKCa[1]**
**Clone**	**Number of unique reads**	**Number of unique reads**	**Number of unique reads**	**Number of unique reads**	**Number of unique reads**	**Number of unique reads**
**1**	258	105	42	109	78	205
**2**	165	41	41	57	77	146
**3**	126	38	41	45	61	113
**4**	75	37	40	43	53	66
**5**	70	30	36	37	38	48
**6**	52	29	36	37	33	38
7	40	27	32	36	31	24
**8**	31	27	28	32	28	23
**9**	25	26	29	31	27	23
**10**	22	24	26	31	27	21

[1]F# - Fish # in group; V - Vaccinated; cluster from K/S - Kidney/Spleen and cluster # - a/b.
Shaded cells are clones of IgZ isotype.

Table S6. Transcripts identified in MMCs by RT-PCR or a transcriptome (NCBI SRA PRJNA852554), that could facilitate germinal center processes.

Gene name	Accession	Description
BAFF	JF798633	Carassius auratus BAFF mRNA, complete cds.
pIgR-like	XM026264707	PREDICTED: Carassius auratus polymeric immunoglobulin receptor-like
pIgR-like	XM026255346	PREDICTED: Carassius auratus polymeric immunoglobulin receptor-like (LOC113086155), transcript variant X3, mRNA.
pIgR-like	XM026255457	PREDICTED: Carassius auratus polymeric immunoglobulin receptor-like (LOC113086587), mRNA
FcγR-like	XM026200605	PREDICTED: Carassius auratus high affinity immunoglobulin gamma Fc receptor I-like (LOC113042005), mRNA.
CR1-like	XM026236798	PREDICTED: Carassius auratus complement receptor type 1-like isoform X2
CR1-like	XM026243819	PREDICTED: Carassius auratus complement receptor type 1-like (LOC113070510), transcript variant X1, mRNA.
MFGE8	XM026202537	Carassius auratus EGF-like repeat and discoidin I-like domain-containing protein 3 (LOC113043284), transcript variant X2, mRNA
CXCL13	CA00064197*	BLAST to PREDICTED: Sinocyclocheilus rhinocerous C-X-C motif chemokine 13-like (LOC107746849), mRNA - XM016561181

* Unannotated goldfish gene accession number from https://research.nhgri.nih.gov/goldfish/

Figure S1

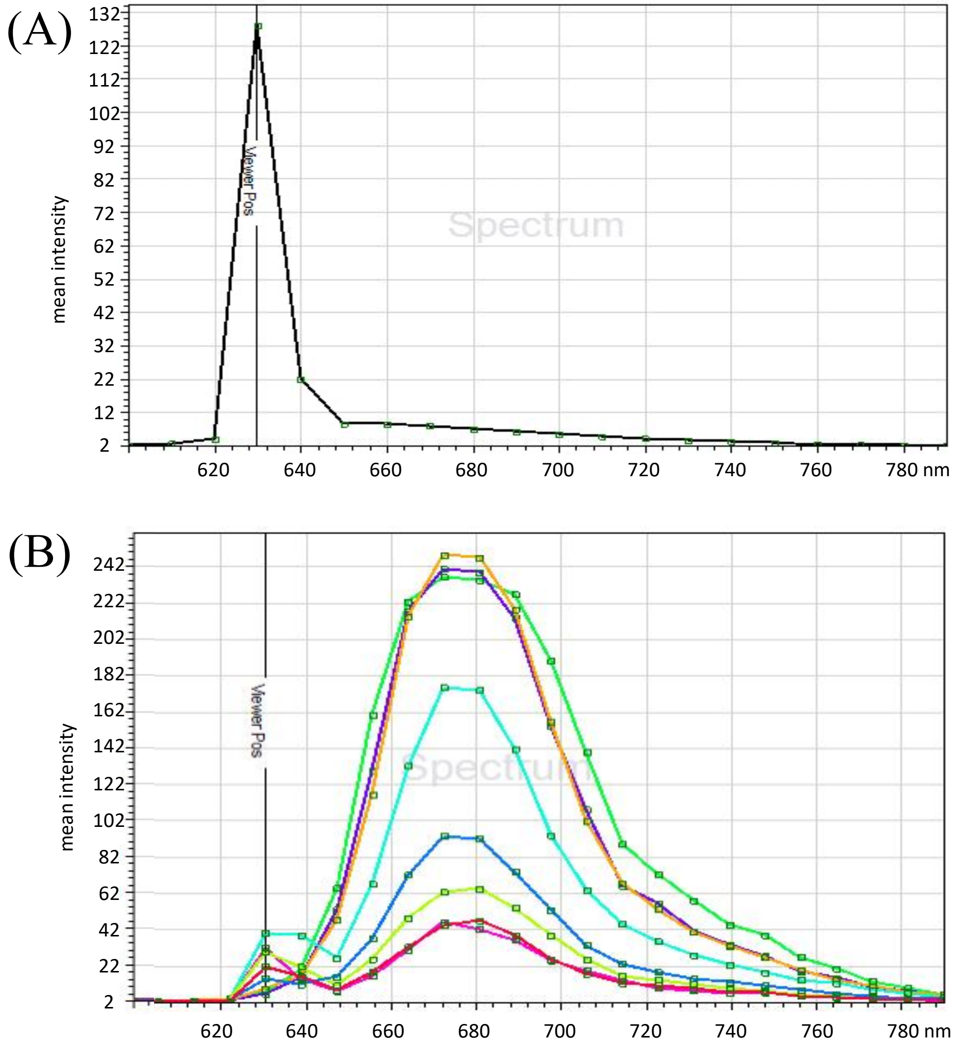

Figure S1 – Laser scanning confocal microscopy Lambda (emission) profiles of individual melano-macrophages exposed to a Cy5 laser (633 nm). A melano-macrophage isolated from the kidney of an unvaccinated goldfish (A), and 8 melano-macrophages (B; separate lines) isolated from the kidney and spleen of a goldfish vaccinated with BSA-Alexa647, and boosted with the same labelled antigen 14 days prior to tissue harvest.

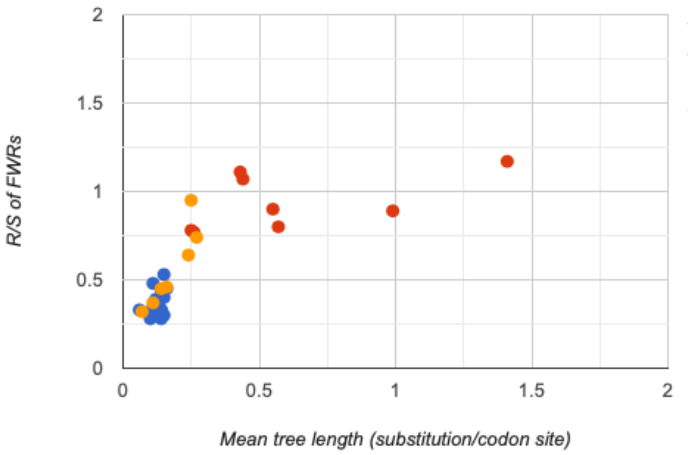

Figure S2. Association between the mean tree length and the ratio of R/S in the FWRs of the Ig repertoires isolated from MMΦCs from unvaccinated and vaccinated zebrafish. Blue - IgM repertoires from MMΦCs from both unvaccinated and vaccinated fish; Red - IgZ repertoires from MMΦCs from unvaccinated fish; Orange - IgZ repertoires from MMΦCs from vaccinated fish.

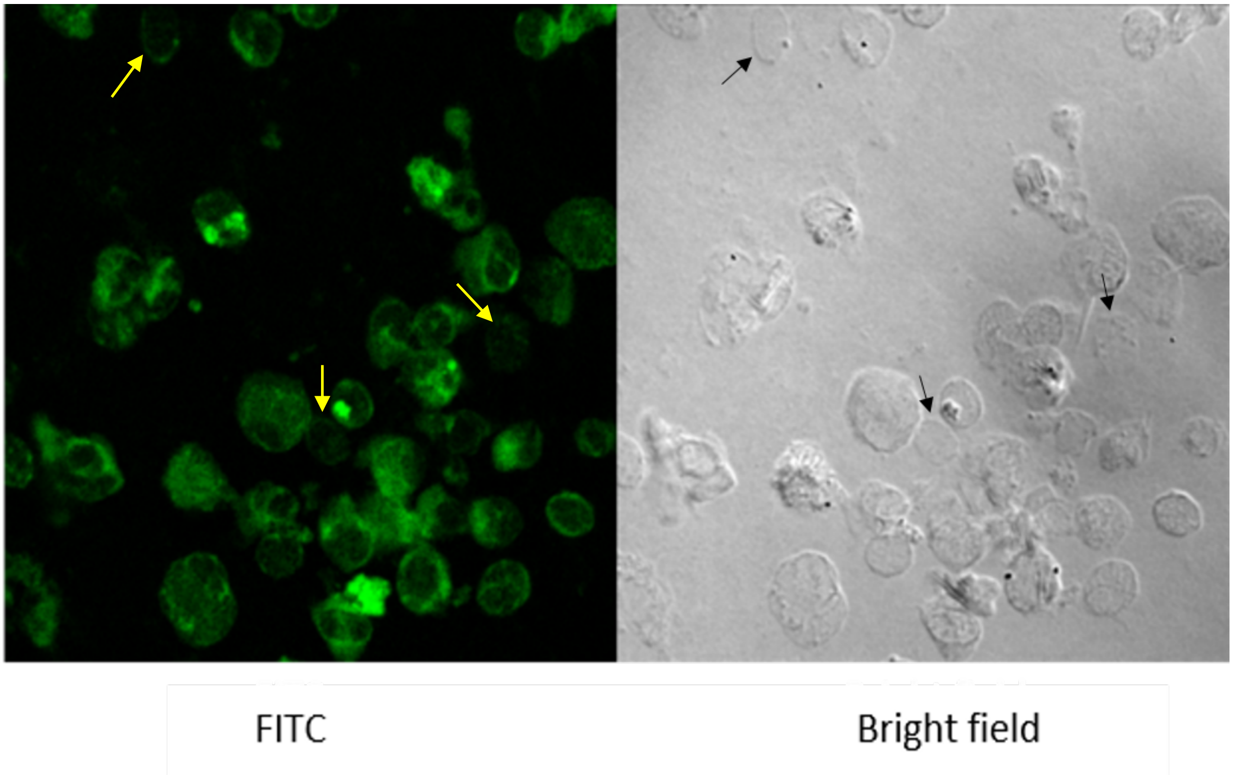

Figure S3. Cells from BSA vaccinated goldfish spleen eluted from beads conjugated with anti-BSA PAb. The vast majority of eluted cells autofluoresce green indicating that they are melanomacrophages. Less mature melanomacrophages (arrows) may have very little accumulated pigment and autofluorescence.
